# Influence of obesity on vertebral fracture prevalence and vitamin D status in postmenopausal women

**DOI:** 10.1186/s12986-015-0041-2

**Published:** 2015-11-14

**Authors:** A. El Maghraoui, S. Sadni, A. El Maataoui, A. Majjad, A. Rezqi, Z. Ouzzif, A. Mounach

**Affiliations:** Rheumatology Department, Military Hospital Mohammed V, Mohammed V University, PO Box: 1018, Rabat, Morocco; Biochemistry Department, Military Hospital Mohammed V, Mohammed V University, Rabat, Morocco

**Keywords:** Vertebral fracture assessment (VFA), Dual-energy X-ray absorptiometry (DXA), Osteoporosis, Women, Vertebral fractures, Vitamin D

## Abstract

**Background:**

It is well established that weight is an important determinant of bone health. Whereas obesity is associated with increased mortality and morbidity from diabetes and cardiovascular diseases, high body weight is widely believed to be associated to hypovitaminosis D and protective against the development of osteoporosis and fracture risk. The objective of the study was to evaluate the effect of BMI on vitamin D status and on densitometric vertebral fractures (VFs) in a large series of asymptomatic women aged over 50 who had a VFA examination during their bone mineral density (BMD) testing.

**Methods:**

We enrolled 429 postmenopausal women (mean age, weight and BMI of 59.5 ± 8.3 (50 to 83) years, 75.8 ± 13.3 (35 to 165) kgs and 29.9 ± 5.2 (14.6 to 50.8) kg/m^2^, respectively. Lateral VFA images and scans of the lumbar spine and proximal femur were obtained using a Lunar Prodigy densitometer. VFs were defined using the Genant semiquantitative (SQ) approach. Clinical risk factors of osteoporosis were collected and 25-hydroxivitamin D was measured using electrochimiluminescence (Roche).

**Results:**

Prevalence of osteoporosis and hypovitaminosis D (<20 ng/ml) was 21.0 % and 78.1 % respectively. VFs grade 2/3were identified in 76 (17.7 %). Comparison between women according to their BMI showed that obese women had a higher BMD and less proportion of women with osteoporosis and VFs grade 2/3 than lean and overweight women. The prevalence of VFs globally increased with age and as BMI and BMD declined. Stepwise regression analysis showed that the presence of osteoporosis was independently related to BMI and history of fractures while the presence of grade 2/3 VFs was independently related to age, hypovitaminosis D and years of menopause.

**Conclusion:**

Obese women had a higher BMD and lower prevalence of VFs. VFs were significantly related to age, hypovitaminosis D and years since menopause. However, among obese women, prevalence of VFs was increased in osteoporotic women.

## Background

Patients with osteoporosis are at increased risk for fractures especially vertebral, wrist, and hip fractures [1]. Vertebral fractures (VFs) can easily go undiagnosed as they are clinically silent in two third of the cases. Moreover, even when they are asymptomatic, they are associated with reduced quality of life, increased morbidity and mortality, and increased risk of future vertebral and non-VFs [2, 3]. Several studies showed that weight is an important determinant of bone health. In early adulthood, weight may determine peak bone mass, and persons who are overweight in young age may be at an advantage regarding bone mass in older age. Thus, whereas obesity is associated with increased mortality and morbidity from diabetes and cardiovascular diseases, high body weight is widely believed to be protective against the development of osteoporosis and fracture risk. The fracture assessment tool FRAX, which includes BMI was developed based on a meta-analysis of 12 cohorts, which showed a negative correlation between BMI and incident hip, osteoporotic, and all fractures, when the models did not include BMD. This finding has been recently confirmed by the Million Women Study [4], a large-scale observational study which found that women with higher BMI have not only increased BMD but also robust femur geometry assessed by hip structure analysis. The negative BMI–fracture association was specific to hip and central body fractures [5]. However, the effect of BMI on fracture at a given level of BMD remains controversial, partly because of its differential effects on different fracture sites. It has been suggested that increased BMI was a protective factor of hip fracture while obesity was a risk factor of ankle and upper leg fractures. Confirmation that obesity was not a protective factor against low trauma fractures was reported in 2011, when the Global Study of Osteoporosis in Women (GLOW), a multicentre prospective observational study conducted in 60,393 postmenopausal women, showed a comparable prevalence and incidence of fractures in normal weight and obese women [6].

The prevalence and clinical risk factors associated with VFs have been well studied in women [7–10]. A negative effect of BMI on VFs was found in a pan-European study in which the prevalence of vertebral deformity in a given country was inversely related to the mean BMI in the study population in that country (*r* = −0.66) [11]. However, Pirro et al. showed that an association between BMI and a higher likelihood of having a VF, irrespective of the positive association between BMI and BMD [12]. We showed in a previous study that VFs prevalence was associated to vitamin D deficiency in a cohort of post menopausal women [13]. On the other hand, vitamin D deficiency has been associated with obesity irrespective of age, latitude, cut-offs to define vitamin D deficiency and the Human Development Index of the study location [14–17]. Thus, we aimed in the present study to evaluate the effect of BMI on vitamin D status and on densitometric vertebral VFs in a large series of asymptomatic women aged over 50 who had a VFA examination during their bone mineral density (BMD) testing.

## Methods

### Subjects

This was a cross-sectional study conducted from June 2012 to June 2014 that enrolled menopausal women 50 years old and over, living in the region of Rabat. Participants were recruited from a variety of sources including advertisements, “word of mouth”, clinician referrals, or from local hospital staff families. General exclusion criteria were previous diagnosis of osteoporosis, non-Caucasian origin and diseases, drugs, and other major determinants known to affect bone metabolism. Thus, we excluded subjects with gastrectomy, intestinal resection, recent hyperthyroidism or hyperparathyroidism, recent severe immobilization or treatment with corticosteroids (more than 3 months). Our institutional review board approved this study. The procedures of the study were in accordance with the Declaration of Helsinki, and formal ethics committee approval was obtained for the study. Data from four hundred and twenty nine were collected. All the participants gave an informed and written consent. Each subject completed a standardized questionnaire designed to document putative risk factors of osteoporosis. History of fractures, lifestyle (alcohol consumption, gymnastics or jogging/walking, smoking) and diet (milk, yogurt, cSSheese) habits were also recorded. The women were asked whether they usually drank milk, coffee, or alcohol, if they ate cheese or yogurt, if they did gymnastics or jogging/walking, and if they smoked tobacco. Dairy products intake was then arbitrarily classified as low (mean calcium intake <600 mg/day), normal (mean calcium intake > 600 mg/day and ≤ 1000 mg/day), and high (≥1000 mg/day). Physical activity was classified as low if the women walked < 3 h/week, normal (walking time ≥ 3 h/week and <6 h/week), and high (≥ 6 h/week). Menstrual and reproductive history were assessed: all patients were menopausal since at least one year. Height and weight were measured in our centre before DXA measurement, in light indoor clothes without shoes. Body mass index (BMI)] was calculated by dividing weight in kilograms by height in meters squared.

### BMD measurement

Bone mineral density was determined by a Lunar Prodigy Vision DXA system (GE-Lunar Corp., Madison, WI). The DXA scans were obtained by standard procedures supplied by the manufacturer for scanning and analysis. All BMD measurements were carried out by 2 experienced technicians. Daily quality control was carried out by measurement of a Lunar phantom. At the time of the study, phantom measurements showed stable results. The phantom precision expressed as the coefficient of variation percentage was 0.08. Moreover, reproducibility has been assessed in clinical practice and showed a smallest detectable difference of 0.040 g/cm^2^ (spine) and 0.021 (total hips) [18, 19]. Patient BMD was measured at the lumbar spine (anteroposterior projection at L1-L4) and at the hip (i.e., femoral neck, trochanter, and total hip). Using the Moroccan female normative data [20], the World Health Organization (WHO) classification system was applied, defining osteoporosis as T-score ≤ −2.5 and osteopenia as −2.5 < T-score < −1. Study participants were categorized by the lowest T-score of the L1–4 lumbar spine, femur neck, or total femur.

### Vertebral Fracture Assessment (VFA)

VFA was classified using a combination of Genant semiquantitative (SQ) approach and morphometry in the following manner: each VFA image was inspected visually by one clinician (AM) who is an experienced reader of VFA which was blinded to clinical data, to decide whether it contained a fracture in any of the visualized vertebrae and assigned a grade based on Genant SQ scale [21], where grade 1 (mild) fracture is a reduction in vertebral height of 20-25 %, grade 2 (moderate) a reduction of 26-40 %, and grade 3 (severe) a reduction of over 40 %. In case of doubt regarding fracture grade, the vertebrae in question was measured using built-in morphometry. Automatic vertebral recognition by the software was used. Positioning of the six morphometry points was modified only when the software failed to correctly recognize vertebral heights. Subjects with no fractures were included in the non-fracture group, whereas those with grade1 or higher fractures were included in the fracture group. However, as many studies rarely report mild deformities as “fractures”, and to realize comparisons with the literature, we performed a double analysis including and excluding grade 1 fractures from the fracture group.

### Laboratory evaluation

All blood samples were collected under fasting conditions on the same day of acquisition of the VFA images, between 8 and 10 a.m., stored at −20 °C, and analyzed at the same time. The serum concentration of 25-hydroxivitamin D (25 (OH) D) was measured using electrochimiluminescence on ELECSYS 2010 analyser (Roche Diagnostics, Mannheim). The intra and inter-assay variation coefficients in our laboratory were 10.5 % and 17.8 %, respectively. In the present study, 25 (OH) D values of ≤ 20 ng/ml were defined as vitamin D insufficiency and of ≤ 10 ng/ml as vitamin D deficiency.

### Statistical analysis

Results are presented as means (SD) and categorical variables are expressed as frequencies. To compare women according to their BMI, women with normal weight, overweight and obesity, chi-square test and analysis of variance ANOVA were used firstly. Women with grade 1 vertebral deformities, women with grade 2/3 VFs and women without VFs were also compared using chi-square test and analysis of variance ANOVA. We performed pairwise comparisons among the 3 groups using the Bonferoni test. Significant risk factors in the univariate analysis for osteoporosis and VFs were entered to a stepwise conditional binary regression analysis and the resulted odds ratios with 95 % confidence intervals were reported. The level for significance was taken as p ≤ 0.05. Excel 2010 and SPSS 20.0 were used for statistical analysis.

## Results

### Patient demographics

In this series of 429 women, the mean ± SD (range) age, weight and BMI were 59.5 ± 8.3 (50 to 83) years, 75.8 ± 13.3 (35 to 165) kgs and 29.9 ± 5.2 (14.6 to 50.8) kg/m^2^, respectively (Table 1). Prevalence of obesity (BMI > 30) was 45.6 % in our population. According to the WHO classification, 90 had osteoporosis (21.0 %) and 252 had osteopenia (58.7 %). None of the women were current smokers while 57 (13.3 %) women reported a history of peripheral fracture after the age of 50. The mean (SD) 25 (OH) D level in this series was 14.5 ng/ml (12.4). Prevalence of hypovitaminosis D was 78.1 % (335/429) for levels < 20 ng/ml and 90.39 (390/429) for levels < 30 ng/ml. Two hundred and twenty four women (52.2 %) had vitamin D deficiency (<10 ng/ml).

**Table 1:** Demographic and clinical parameters of participants (*n* = 429)

			Minimum	Maximum
Age (yrs) : m (SD)	59.5	(8.3)	50	83
Height (cm) : m (SD)	156.5	(6.1)	138	185
Weight (Kg) : m (SD)	75.8	(13.3)	35	165
BMI (kg/m^2^) : m (SD)	29.9	(5.2)	14.6	50.8
Years since menopause (yrs): m (SD)	9.7	(8.9)	1	50
Number of pregnancies: m (SD)	5.2	(2.3)	0	13
History of fractures: n (%)	57	(13.3)		
Lumbar spine BMD (g/cm^2^): m (SD)	0.967	(0.19)	0.641	1.413
Total hip BMD (g/cm^2^): m (SD)	0.834	(0.43)	0.583	1.460
Lumbar spine T-score: m (SD)	−1.5	(1.3)	−5.2	4.00
Total hip T-score: m (SD)	−1.01	(1.2)	−4.7	3.02
Osteoporosis any site : n (%)	90	(21.0)		
Vitamin D (ng/mL) : m(SD)	14.5	(12.4)	3	116
Vitamin D < 20 ng/mL : n(%)	335	(78.1)		
Vitamin D < 10 ng/mL : n(%)	224	(52.2)		
VFs grade 2/3: n (%)	76	(17.7)		

*BMI* body mass index, *BMD* bone mineral density

### Vertebral visualization and fracture identification on VFA

In these 429 women, 62.7 % of vertebrae from T4–L4 and 99 % from T8–L4 were adequately visualized on VFA. The percentage of vertebrae not visualized at T4, T5, and T6 levels was 57.1 %, 35.2 %, and 20.1 % respectively.

VFs were identified in 248 (57.8 %): 172 (40.1 %) had grade 1 and 76 (17.7 %) had grade 2 or 3. Fractures were most common in the mid-thoracic spine and at the thoracolumbar junction.

Comparison between women according to their BMI showed that obese women had a higher lumbar spine and total hip BMD and less proportion of women with osteoporosis and VFs grade 2/3 than lean and overweight women (Table 2).

**Table 2:** Comparison between women according to BMI (*N* = 429)

	BMI ≤ 25	25 < BMI ≤ 30	BMI > 30	
	*N* = 75	*N* = 158	*N* = 196	
Age in years: m (SD)	59.1 (7.9)	59.1 (7.9)	58.7 (7.7)	NS
Weight in Kg: m (SD)	60.0 (10.1)	70.3 (9.8)	84.9 (8.3)	<0.0001
Height in m : m (SD)	1.57 (0.08)	1.57 (0.05)	1.55 (0.05)	<0.0001
BMI in Kg/m2: m (SD)	22.9 (1.5)	27.5 (1.3)	34.8 (1.4)	<0.0001
Years since menopause: m (SD)	10.2 (8.8)	9.4 (9.3)	9.6 (8.3)	NS
Number of pregnancies: m (SD)	5.0 (2.7)	4.6 (2.5)	4.9 (2.3)	NS
History of traumatic fractures	13 (17.3)	16 (10.2)	28 (14.7)	NS
Low dairy products intake: n (%)	32 (45.1)	75 (47.8)	97 (51.3)	NS
Low physical activity: n (%)	65 (86.6)	137 (86.7)	172 (87.7)	NS
Spine BMD in g/cm^2^: m (SD)	0.835 (0.2)	0.951 (0.1)	1.022 (0.2)	<0.0001
Spine T-score: m (SD)	−2.4 (1.3)	−1.4 (1.2)	−0.8 (1.3)	<0.0001
Total hip BMD in g/cm^2^: m (SD)	0.808 (0.2)	0.855 (0.5)	0.913 (0.4)	0.011
Total hip T-score: m (SD)	−1.7 (1.1)	−1.1 (1.0)	−0.5 (1.1)	<0.0001
Osteoporosis any site : n (%)	31 (41.3)	38 (24.9)	20 (10.0)	<0.0001
Grade 1 VFs prevalence (%)	28 (37.3)	55 (34.8)	86 (45.3)	NS
Grade 2/3 VFs prevalence (%)	17 (22.3)	33 (20.9)	26 (13.7)	<0.0001
SDI: m (SD)	0.54 (2.1)	0.92 (2.4)	0.51 (1.9)	NS
Vitamin D in ng/mL: m (SD)	12.9 (9.1)	14.4 (11.6)	15.9 (13.2)	NS
Vitamin D ≤ 20 ng/mL: n (%)	58 (77.3)	122 (77.8)	149 (78.4)	NS
Vitamin D ≤ 10 ng/mL: n (%)	42 (56.0)	80 (50.8)	99 (50.5)	NS

*BMI* body mass index, *BMD* lumbar spine BMD, *SDI* spinal disease index

The group of women with moderate/severe VFs had a statistically significant higher age, number of parity, years since menopause and lower weight, height, and lumbar spine and total hip BMD and T-scores than those without a VFA-identified vertebral fracture (Table 3). They also have lower levels of 25 (OH) D and higher proportion of women with vitamin D below 20 ng/ml.

**Table 3:** Comparison between women with and without vertebral fractures (*N* = 429)

	Absence of VFs	VFs grade 1	VFs grade 2/3	
	*N* = 181	*N* = 171	*N* = 76	
Age in years: m (SD)	56.1 (7.9)	60.1 (7.9)	64.7 (7.7)	<0.0001
Weight in Kg: m (SD)	76.0 (10.1)	75.3 (9.8)	70.9 (8.3)	<0.0001
Height in m: m (SD)	1.57 (0.08)	1.55 (0.05)	1.56 (0.05)	<0.0001
BMI in Kg/m^2^: m (SD)	30.0 (1.5)	30.2 (1.3)	28.8 (1.4)	<0.0001
Low dairy products intake: n (%)	99 (54.6)	72 (42.1)	39 (51.3)	NS
Low physical activity: n (%)	165 (91.1)	148 (86.5)	72 (91.1)	NS
History of traumatic fractures: n (%)	22 (12.2)	22 (12.9)	13 (17.1)	NS
Years since menopause: m (SD)	6.8 (8.8)	10.4 (9.3)	16.6 (8.3)	<0.0001
Number of pregnancies: m (SD)	4.5 (2.7)	4.8 (2.5)	5.5 (2.3)	0.015
Spine BMD in g/cm^2^: m (SD)	0.987 (0.2)	0.923 (0.1)	0.846 (0.2)	0.025
Spine T-score: m (SD)	−1.1 (1.3)	−1.3 (1.2)	−1.5 (1.3)	0.049
Total hip BMD in g/cm^2^: m (SD)	0.921 (0.2)	0.850 (0.5)	0.801 (0.4)	0.011
Total hip T-score: m (SD)	−0.7 (1.1)	−1.0 (1.0)	−1.2 (1.1)	0.004
Osteoporosis any site : n (%)	25 (13.8)	42 (24.4)	23 (30.3)	<0.0001
Vitamin D in ng/mL : m (SD)	16.2 (9.1)	14.2 (11.6)	11.5 (13.2)	0.02
Vitamin D ≤ 20 ng/mL: n (%)	133 (73.5)	130 (75.6)	72 (94.7)	0.001
Vitamin D ≤ 10 ng/mL: n (%)	12 (34.3)	59 (57.3)	29 (65.9)	0.006

*BMI* body mass index, *BMD* lumbar spine BMD

In this study, VFA-identified fractures occurred in 145 (57.1 %) women with osteopenia (44 (17.5 %) had grade 2/3) and in 65 (71.9 %) women with osteoporosis (23 (25.6 %) had grade 2/3) (*p* < 0.0001). Interestingly, a fracture was identified on VFA in 38 (43.6 %) of women with normal BMD (9 (10.3 %) had grade 2/3 VFs).

Among obese women (*n* = 210 (49.1 %)), VFs prevalence was higher in those with osteoporosis (23.1 %) vs. those with osteopenia (12.0 %) and normal BMD (4.5 %) (*p* < 0.0001). The prevalence of hypovitaminosis D was also higher even though the difference was not significant (100 %, 90.0 % and 54.5 % respectively; *p* = 0.34)

### Risk factors

The prevalence of VFA-detected fractures globally increased significantly for both grade 1 and grade 2/3 VFs with age (*p* < 0.0001) and as BMD (*p* < 0.0001) and BMI declined (*p* < 0.0001).

Stepwise regression analysis showed that presence of osteoporosis was independently related to BMI and history of fractures (Table 4) while the presence of VFs grade 2/3 was independently related to age, hypovitaminosis D and years of menopause (Table 5).

**Table 4:** Regression logistic analysis with the presence of osteoporosis as the dependent variable

	OR total population	p
Age in years	0.976 (0.927–1.077)	0.351
BMI in Kg/m^2^	0.847 (0.794–0.903)	<0.0001
History of fractures	2.188 (1.072–4.465)	0.031
Vitamin D ≤ 20 ng/mL	1.485 (0.767–2.877)	0.241
Number of pregnancies	1.051 (0.953–1.158)	0.319

*BMI* body mass index

**Table 5:** Regression logistic analysis with the presence of grade 2/3 VFs as the dependent variable

	OR total population	p
Age in years	1.204 (1.103–1.315)	<0.0001
Vitamin D ≤ 20 ng/ml	6.688 (2.244–19.937)	<0.0001
Menopause duration in years	1.053 (1.009–1.098)	0.017
BMI in Kg/m^2^	0.975 (0.914–1.039)	0.435
Lumbar spine T-score	1.085 (0.831–1.415)	0.549
Total hip T-score	0.868 (0.642–1.174)	0.358

*BMI* body mass index

## Discussion

This study is a large descriptive evaluation of VFA in a population of asymptomatic postmenopausal women and documents that VFs are significantly related to age, hypovitaminosis D and years of menopause while the influence of BMI was only evident on BMD.

About 18 % of asymptomatic healthy women over 50 had a previously undiagnosed grade 2/3 VFs. This prevalence varied according to BMI and was higher in lean and overweight groups (22 and 21 % respectively compared to obese women (13 %). However, among obese women this prevalence was 23 % in osteoporotic women.

Obesity, defined by the World Health Organization (WHO) as a body mass index (BMI) ≥30 kg/m^2^, has become a widespread disease. According to WHO, worldwide prevalence has been steadily increasing to epidemic proportions and it has more than doubled worldwide since 1980 [22]. Obesity is associated with high BMD [23], while weight loss is associated with bone loss [24]. The mechanisms responsible for this fat-bone relationship are not fully clarified, but weight change may act on the skeleton through changes in mechanical loading and changes in hormone regulation on bone metabolism. Several studies described the association of fat mass with the secretion of bone active hormones from the pancreatic beta cell (including insulin, amylin, and preptin) and from the adipocyte (e.g., estrogens and leptin) [25–27]. These factors alone probably do not fully explain the observed clinical associations, and study of the actions on bone of novel hormones related to nutrition is an important area of further research. Weight changes are also related to changes in lifestyle such as physical activity and intake of nutrients [28].

It is still not clear whether obesity-related higher BMD increases bone strength and thereby protects against fractures and osteoporosis. The effect of obesity on fractures prevalence may vary by fracture type. Two studies demonstrated increased risk of lower limb fractures among obese women, but no increased risk with fractures of other types [5, 29]. According to a large Spanish study, obesity increased the risk of proximal humerus fractures, whereas hip and pelvic fractures were found to be less prevalent [30]. These differences may not be related to BMD but to weight determined higher impact of falls on the peripheral skeleton, whereas other skeletal regions are protected by subcutaneous fat deposits. Some studies support this site-dependent fracture risk both in obese pre- and postmenopausal women and men [31, 32]. Concerning VFs, Laslett LL et al. showed a dose–response relationship between both prevalence and number of vertebral deformities and multiple measures of body fat in women [33]. Ferrar et al. suggested that some types of mild deformities may be “non-fracture variants” rather than vertebral deformities. These include short vertebral height, which they report as being more common in older, heavier women, unrelated to osteoporosis equally prevalent in pre and postmenopausal women and therefore unimportant [34]. The assessment of prevalent mild VFs is still problematic, as such deformities may reflect old trauma or are sometimes considered as an expected effect of aging [35]. Actually this point is raised when such deformities are used to discriminate patients with or without osteoporosis [36]. However, mild VFs (grade 1) have been shown to be a risk factor for subsequent vertebral and non-vertebral fracture in postmenopausal women with osteoporosis [37].

Vitamin D insufficiency may lead to secondary hyperparathyroidism resulting in greater bone turnover, bone loss, and increased risk of fractures. This insufficiency may result from decreased cutaneous synthesis, decreased dietary intake, impaired hepatic and/or renal activation, or resistance to vitamin D action [38]. Serum 25-hydroxyvitamin D (25 (OH) D) level is considered to be the best indicator of vitamin D nutritional status and it has been suggested that serum 25 (OH) D values <10 ng/ml indicate vitamin D deficiency and <20 ng/ml indicate vitamin D insufficiency [39]. Although this was not the case in our study, recent evidence indicates that obesity is linked to lower serum levels of 25 (OH) D [14, 40, 41], which may be due to poor exposure to sunlight and/or decreased vitamin D bioavailability (due to the sequestering effect of high quantity of subcutaneous fat on circulating vitamin D).

Our study has strengths and limitations. The assessment of fracture was carefully conducted using standard procedures of acquisition, and standard reading of all VFAs. All the morphometric assessments were made by an experienced investigator. Before diagnosis of fracture, a non-osteoporotic origin was considered for each deformity. The main limitation lies in the procedures used to select subjects, who were all volunteers and ambulatory, and presumably not representative of the general population. However, the population of Rabat is a mixture of the Moroccan population and we believe that there is a little impact on the prevalence of obesity, osteoporosis or VFs or hypovitaminosis D.

## Conclusion

In a large sample of Moroccan postmenopausal ambulatory women aged 50 years and over, we found that 18 % of asymptomatic women show evidence of moderate/severe VFs and that these VFs were significantly related to age and hypovitaminosis D. We also found that obese women had a higher BMD and lower prevalence of VFs. However, the prevalence of VFs in obese women was similar to non obese women in those with osteoporosis.
